# Fever of Dentition

**Published:** 1878-10-20

**Authors:** 


### FEVER OF DENTITION.

A writer (in Dental cosmos) on the high fevers and nervous excitement of teething children, threating spasm says:

As means to allay nervous and arterial excitability, no medicine in the experience of the writer, surpasses the bromide of potassium, or a combination of that salt with veratrum viride.

To one or two drops of Norwood's tincture of veratrum viride the writer conjoins five to ten grains of bromide of potassium, repeating the dose every two, three, or four hours, as found necessary. If irritability of stomach exists, the bromide of camphor may take the place of the potassium.

As formulæ for the first, the following may be given:

R.—Tinct. verat. viride (Norwood's,) gtt. viii;

Potass. bromidi, one drachm;

Aquæ, one ounce M.

A teaspoonful pro re nata.

In using the veratrum viride it may be best to employ it unmixed with other medicines,—that is, where a nurse is to be trusted. Given in drop doses, it can be repeated each half hour, or hour, until the frequency of pulse is found to yield. It is, however, a formidable agent, and is not to be used except under close supervision by the physician.

Lancing the tumid and conjested gums is a practice that, in the majority of cases will anticipate all necessity for constitutional medication. A lancet and an aperient, or a little iced lemonade, constitute the therapy commonly demanded in practice.

Concerning irritability engendered in the spinal cord by improper motions, much is to be said.

At the present time the writer has under his professional care a patient, where spasms of the neck-muscles of a most unyielding character are traceable directly to concussion produced from riding over the paved streets, although the carriage used by the lady is of the most luxurious description.

The jumping up and down of a baby in the arms of a nurse is, at the dentitional period, a reprehensible practice; quiet is the indication.

Not always is it, however, that dentitional irritability is found to have associated with it vascular perversion. It is grave to commit fault in such direction of diagnosis. To make such mistake could have no excuse; to learn the condition implies alone the placing of a finger upon the temporal artery, together with appreciation of collateral circumstances.—A. B. C.

